# From silos to synergy: integrating academic health informatics with operational IT for healthcare transformation

**DOI:** 10.1038/s41746-024-01179-5

**Published:** 2024-07-09

**Authors:** Devin M. Mann, Elizabeth R. Stevens, Paul Testa, Nader Mherabi

**Affiliations:** 1 Department of Population Health, NYU Grossman School of Medicine, New York, NY USA; 2 MCIT Department of Health Informatics, NYU Langone Health, New York, NY USA

**Keywords:** Institutions, Health services

## Abstract

We have entered a new age of health informatics—applied health informatics—where digital health innovation cannot be pursued without considering operational needs. In this new digital health era, creating an integrated applied health informatics system will be essential for health systems to achieve informatics healthcare goals. Integration of information technology (IT) and health informatics does not naturally occur without a deliberate and intentional shift towards unification. Recognizing this, NYU Langone Health’s (NYULH) Medical Center IT (MCIT) has taken proactive measures to vertically integrate academic informatics and operational IT through the establishment of the MCIT Department of Health Informatics (DHI). The creation of the NYULH DHI showcases the drivers, challenges, and ultimate successes of our enterprise effort to align academic health informatics with IT; providing a model for the creation of the applied health informatics programs required for academic health systems to thrive in the increasingly digitized healthcare landscape.

We have entered a new age of health informatics. The ubiquitous and rapidly evolving use of technology in healthcare demands that institutions adapt to integrate operational and innovative academic informatics resources. Historical organizational models of health informatics that silo academic health informatics departments from operational information technology (IT) hinder a health system’s ability to adapt, scale, and respond to new technological developments required for achieving healthcare goals. As the science of using data, information, and knowledge to improve human health and healthcare services, health informatics plays an ever more critical role in how healthcare is delivered. To fulfill these roles, the field of health informatics has dynamically adapted to the growing digitization of healthcare. Nevertheless, for health informatics to address our healthcare systems’ current and future requirements, it must undergo further evolution. This evolution requires direct intervention and concerted, collaborative efforts from health systems and academic health informaticists. By showcasing the successes and challenges of our institution’s ongoing endeavors to align academic health informatics with IT, we aim to underscore the necessity for integrating health informatics programs with IT and present this model for others to consider when developing their own strategies to address the needs of this new age of health informatics. This new integration model is crucial to effectively respond to the technological demands of healthcare, both now and in the future.

## An evolution of health informatics

Health informatics, having grown up in a classic academic tradition—i.e., built by researchers at academic institutions with external research funding (such as from the National Institutes of Health) with the explicit purpose of creating knowledge—has often been steered towards the aspirational goal of indirectly influencing healthcare delivery and human health by providing new tools and evidence for healthcare decision makers and providers^1^. With the digitization of healthcare itself, accelerated through federal policies such as the HITECH Act^2^, informatics became critical to health system operations, generating the growth of the previously undefined “operational informatics.” Thus, a hybrid system was created where classic informatics and operational informatics have worked in parallel—with opportunistic collaboration—generally remaining siloed from each other.

Informaticists within academic healthcare institutions have frequently been separated from IT teams—and do not even exist in non-academic settings. IT teams manage the hardware and software required to run hospital clinical systems and support other health system functions such as education and research (e.g. statistical software, basic science hardware/software, library systems, etc.). Alternatively, informaticists have been tasked with focusing on more conceptual innovation and research to develop new digital health tools and systems^3^. However, with the widespread adoption of enterprise electronic health records (EHRs) and steps being taken towards digitizing medicine, this compartmentalization has become unsustainable. As a result, IT and informatics teams have been impelled to intersect in more meaningful and pragmatic ways^4^; however, significant barriers to these collaborations remain without unified efforts to facilitate these intersections.

Against this backdrop, we have seen the emergence of a new age of informatics—applied health informatics—where digital health innovation cannot be pursued without considering operational needs^5^. Within many institutions, actions have been taken to address the need for consideration of clinical care operations within IT, with the creation of positions such as the Chief Medical Information Officer (CMIO), however, additional strategies are needed to address the challenges faced in within more academic-oriented informatics. Seemingly paradoxically, with the rise of digitization in operational settings, informaticists have seen more significant restrictions to accessing data and implementing novel tools into clinical care for their research. EHRs and the digitization of medicine dramatically elevated the consequences and financial implications of IT, where health systems that leveraged digital tools well were at an advantage compared to their peers; leading to increased guarding of IT resources^6^. Furthermore, the interconnectivity of health IT systems has led to fewer interventions being isolated. Even straightforward and effective tools, such as clinical decision support alerts, have rapidly become overwhelming, resulting in issues like alert fatigue that carry significant consequences at a health system level^7^.

This new reality has enhanced scrutiny by operational and IT leadership with concomitant growth of operational IT programs as a necessity in facilitating academic informatics research. It is now often the case that minimal informatics research can be performed without health system leadership oversight and approval. IT teams have become the new stewards to the treasure trove of informatics research enabled by the EHR and other dimensions of digital medicine applied to innovative digital health research studies, including patient portals, smartphones, and apps. In many academic medical centers, this gatekeeper dynamic has created an undefined and inconsistent partnership between IT and informatics teams that is likely to prove untenable moving into the future. In this new digital health era, where applied health informatics will predominate, creating an integrative system—where a true partnership is established to join operational and academic informatics people, resources, and missions—will be essential for health systems to achieve informatics healthcare goals effectively. Indeed, many calls have been made to create processes that unite informatics innovation with operational IT^8–10^, and steps have been taken to accomplish this goal, including creating positions such as Chief Research Informatics Officers (CRIOs)^11^, however further actions are needed to develop systems that represent a structural integration between informatics research and operational IT.

## Developing an integrated system

In this new era, there is a fundamental need for ongoing experimentation and innovation through researchers’, IT’s, and operational leadership’s collaborative efforts. Achieving this necessitates an optimized organizational infrastructure, which can be realized by fully integrating IT and health informatics. While it might be expected that this integration would naturally occur in response to technological advancements, without a deliberate and intentional shift towards unification, operational IT and health informatics convergence is unlikely to happen successfully. Such unification is essential for effectively addressing the constantly evolving landscape of health technology^8^. Recognizing this, NYU Langone Health’s (NYULH) Medical Center IT (MCIT) executed a strategic plan and intentional, collaborative system-level effort to create an integrated system that allows for advancement in real-world applications, as well as innovation at scale. This endeavor united MCIT and academic health informatics, giving operational and academic innovation teams access to the wealth of health informatics expertise and greater freedom to experiment. This deliberate restructuring has already begun to show benefits as new disruptive technologies—like generative artificial intelligence (GenAI)—have emerged, and the efforts made by NYULH may serve as a roadmap for other health systems seeking a strategy to advance their own capabilities to adapt to rapidly emerging technological changes.

As with many academic health systems, historically, NYULH MCIT and health informatics were fundamentally unrelated endeavors. Despite successfully addressing the current technological needs of a digitizing health system through progressive strategies, such as transitioning from an application-based structure to an experience-based one (i.e., project portfolios centered on the user rather than the technical system), these restructuring efforts did not fully integrate MCIT and informatics. In this context, there was a recognition that maintaining the status quo and relying on incremental changes to legacy structures that governed the relationship between IT and health informatics would constrain NYULH’s ability to maintain excellence across its tripartite mission.

**Fig. 1:**
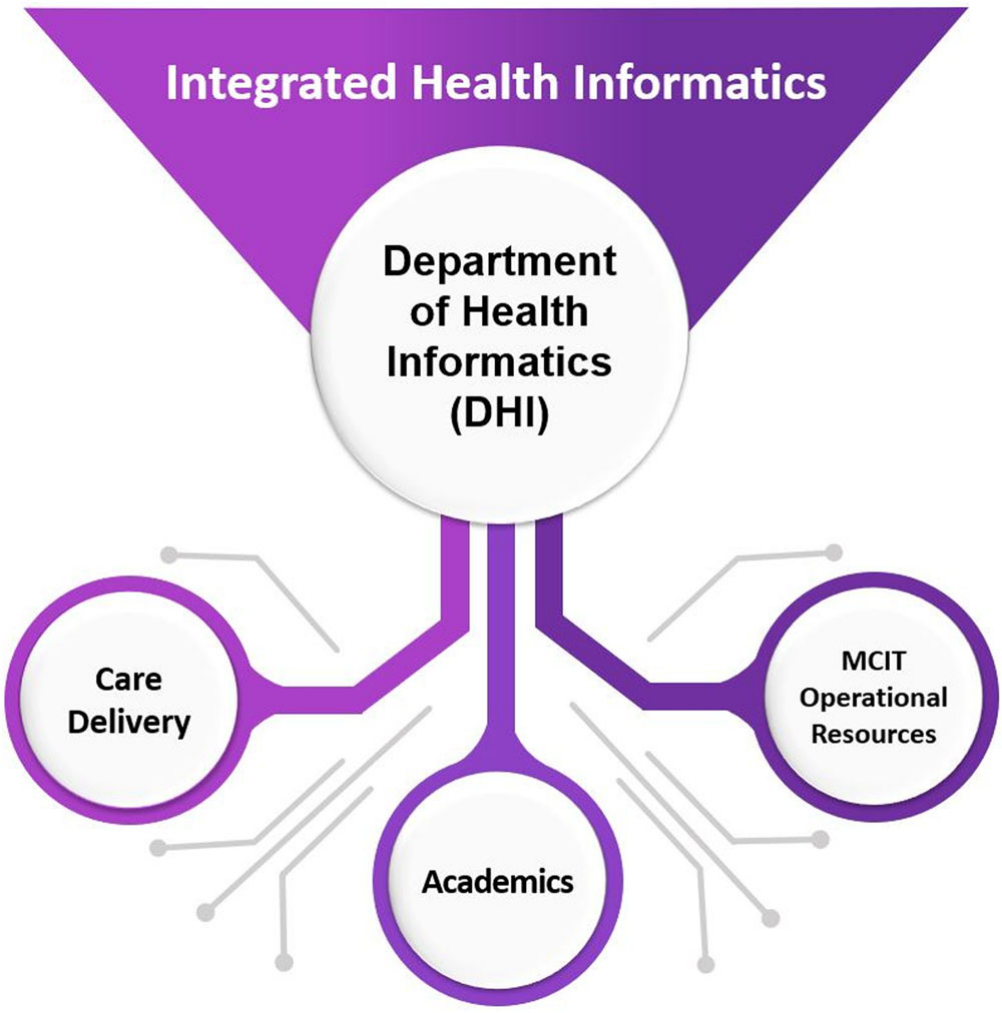
Diagram of the integration of health system mission through the Department of Health Informatics. The Department of Health Informatics creates a new model to promote collaboration for integrated informatics by bringing together stakeholders in care delivery, academics, and operational resources.

The early stages of this transformation began more than a decade ago with incremental reorganization from exclusively IT application-based teams (clinical, hardware, security, etc.) to include joint structures based on shared goals around a theme e.g., a digital experience-based portfolio (patient digital experience, clinician digital experience, researcher digital experience, etc.). This shift reflected the transition to integrated digital experiences and the reorganization of MCIT’s work to better align with its fundamental roles in a large academic health system. These early steps set the stage for the recent full vertical integration of operational IT and health informatics with the NYULH MCIT Department of Health Informatics (DHI) launch in 2023. Establishing DHI within MCIT was unconventional and strategically disruptive. Unlike traditional biomedical or health informatics departments, MCIT DHI is a corporate operations department within IT, not an academic department. It does not confer academic promotion nor “own” faculty or grants—instead, it serves as an enterprise-level hub of informatics activity that spans the full breadth of the health system’s missions—clinical care, research, and education (Fig. 1). This hub integrates the domains of a CMIO and CRIO bringing together clinical and technical operational personnel, researchers, educators, and clinicians seeking to leverage technology to drive scaled innovation across the enterprise. Through this informatics structural innovation, the legacy silos between academic and operational entities within an academic health system were mitigated, amplifying the impact of informatics on clinical operations and research.

The DHI comprises eight divisions—Clinical Informatics, Health IT Safety, Digital Health Innovation, Digital Health Equity, Applied AI, Research Informatics, Nursing Informatics, and Educational Informatics. However, these divisions are not formal entities like the divisions of an academic department; rather, they serve as concentration areas within a matrixed organizational structure^12^. Within this matrixed structure, all core DHI faculty play multiple roles, serving IT operational roles that complement their informatics and academic roles. For example, the Director of the Division of Health Equity in DHI spearheads institution-wide efforts to leverage digital health tools in support of enterprise equity goals across all missions (clinical, research, education, community); a role that jointly reports to (and is partially funded by) the CMIO in MCIT and the Director of the NYULH Institute for Excellence in Health Equity (an enterprise-wide initiative). She is also a tenure-track physician investigator studying clinical decision support tools in the NYULH Department of Population Health (her academic home). Acting as foci of common work, the Divisions do not have their own prioritization structures or committees, instead using established decision-making bodies to facilitate knowledge sharing, remove redundancy and coordinate efforts horizontally across potential silos.

This matrixed role underscores the deliberate nature of a fully integrated health informatics initiative within an academic health system. Consistent with this approach, IT resources and staff are not assigned to specific divisions. Instead, the DHI and Divisions request and get assigned corporate IT resources (EHR analysts, reporting resources, software architecture resources) as needed. They are prioritized in the same enterprise prioritization processes that all IT requests get reviewed. Similarly, non-DHI academic faculty and staff are recruited to lend expertise to DHI initiatives. They are provided an official channel to raise their health informatics research for support from DHI. This allows projects to be easily worked on by multiple teams and divisions organized around the user experience they seek to improve at scale.

## Adaptability in a new technological age

By putting health informatics within an enterprise IT department and creating vertical integration, the unique and intentional structure of the NYULH DHI reflects the needs of academic healthcare systems in the current age. New technological innovations frequently have implications in broad domains. Restructuring how informatics and IT interact within an organization can avoid the resource waste and stifling of progress that can arise by approaching the use of these technologies in a siloed, piecemeal fashion. An integrated system also allows for better measurement of resource use and evaluation of implementation impact. Furthermore, with an increasing number of regulations placed on digital health solutions, implementing academic health informatics as part of operational IT can reduce the challenges associated with system compliance monitoring. Conversely, the creation of DHI has generated challenges and exposes our organizations to potential “threats.” Integrating informatics research, operations and education creates potential role and responsibility confusion, an organizational disruption that requires attention and iterative adjustments and organizational patience as people and teams adapt to the new structures. Additionally, even though DHI is designed to not compete with established organizational structures, leadership needs to be attuned to the risk that over time DHI could develop its own “walls” that create new siloes rather than breaching old ones. Integrating operational and academic IT also highlights the differences in standards, cultures, and other norms between these traditions. For example, while auditable standards may be required for operational IT systems, academic innovations often have less stringent standards. Operational innovation sandboxes^13^ and other approaches to create reduced risk innovation zones within an academic health system represent potential avenues for bridging these differences.

Generative AI (GenAI) exemplifies the fundamental advantages of this organizational approach. This disruptive technology has broad applications within an academic medical center, including in research, education, and clinical care delivery. It is also complex and rapidly evolving, requiring significant expertise, oversight, and resources to implement effectively and safely. Furthermore, due to its very nature, GenAI becomes a more powerful tool when it is integrated with data and additional applications. In a traditional structure, innovations using GenAI would be siloed between the various health system functions, with separate efforts occurring for academic investigations, clinical operations improvement projects, and explorations of its value in medical education.

The DHI leveraged its role as an organizational hub to rapidly establish the first patient health information (PHI) safe OpenAI GPT-4 environment for organizational experimentation, host the first healthcare “prompt-a-thon”, rapidly implement strategically aligned pilots in all of its missions and deploy a unified oversight of GenAI applications^14^. Moreover, the integrated structure facilitates remarkable transparency and transferability of ideas and expertise across domains. This allows IT resources to be more efficiently deployed in support of experimenting with how this important new technology can help drive forward all the academic health system’s missions. Thus, while the creation of a new structure within MCIT has taken significant vision and labor to implement, in the long-term, the effective reorganization and incorporation of health informatics and IT is ultimately reducing the costs associated with implementing these novel and broadly reaching technologies.

## Summary

New technological innovations, such as GenAI, present both exciting opportunities and challenges for health systems to revolutionize their capabilities at scale in all aspects of their missions. The rapid evolution of these technologies necessitates an organizational structure that integrates traditional IT and health informatics into a symbiotic system, fostering innovative ideas, deploying novel clinical tools, and translating research into practice. NYULH is a model for achieving this mutual collaboration and may inspire other organizations. Most importantly, the example of NYULH DHI highlights the significance of strategic planning and deliberate efforts on the part of health system leadership to bring together IT and informatics resources to tackle the challenges that will arise in the new AI-driven era of healthcare innovation.

### Reporting summary

Further information on research design is available in the Nature Research Reporting Summary linked to this article.

### Supplementary information

Reporting Summary
